# Editorial: Antidepressant Prescriptions in Children and Adolescents

**DOI:** 10.3389/fpsyt.2020.600283

**Published:** 2020-10-30

**Authors:** Michael P. Hengartner

**Affiliations:** Department of Applied Psychology, Zurich University of Applied Sciences, Zurich, Switzerland

**Keywords:** antidepressant, pediatric, prescribing, children, adolescents, efficacy, safety, suicidality

The use of antidepressants in children and adolescents has a troubled history, for almost all principles of good evidence-based medicine were violated or compromised. It is a history characterized by systematically biased research, financial conflicts of interest, and professional recklessness (1–3). In 2004, the Lancet Editors (4), in an article titled “Depressing research” bluntly stated that “The story of research into selective serotonin reuptake inhibitor (SSRI) use in childhood depression is one of confusion, manipulation, and institutional failure” (p. 1335). It is now well-established that most pediatric antidepressant trials were industry-sponsored and had serious methodological limitations; many trials remained unpublished due to unfavorable results, and those published were mostly ghost-written, selectively reported efficacy outcomes and misrepresented the true rate of treatment-emergent suicidal events (5–9). Drug regulators issued a suicidality warning for pediatric antidepressant use in 2003 (MHRA) and 2004 (FDA) and advised to use fluoxetine only. By consequence, some authors argued that SSRI should be reserved as a second-line option for youth with severe and resistant forms of depression (10).

However, in most countries antidepressant use has considerably increased in children and adolescent over the last 10–15 years (11–13), despite suicidality warnings, the serious limitations of the evidence-base (14), and ongoing controversies surrounding risks and benefits (15) as well as the placebo response (16). The aim of this special topic was thus to provide a collection of articles broadly focused on two main issues; first, on the current scientific evidence for the efficacy and safety of antidepressants, with a special emphasis on suicidality and related regulatory warnings, and, second, on recent trends in prescription rates and patterns of utilization, including antidepressant overuse, and the increasingly medicalized approach to mental health.

Safer and Zito reviewed the efficacy of new-generation antidepressants for pediatric depression. They found no meaningful benefits in children and only marginal benefits in adolescents based on placebo-controlled short-term trials. Moreover, they considered the evidence for maintenance treatment based on discontinuation (placebo-substitution) trials problematic and inconclusive due to high dropout rates, potential withdrawal syndromes that mimic relapse, and relapse rates not dissimilar from the natural course of the disorder.

Boaden et al. conducted a meta-review on the efficacy, tolerability, and suicidality-risk of antidepressants for the treatment of various pediatric disorders. The meta-review found that just a few antidepressants were effective and well-tolerated. For instance, only fluoxetine was more effective than placebo in major depression, and only fluvoxamine and paroxetine were effective in anxiety disorders. Venlafaxine (in major depression) and paroxetine (in anxiety disorders) were associated with significantly increased risk of suicidality. However, of the nine meta-analyses included, only one met criteria of high quality; five were rated moderate quality, one was of low quality and two of critically low quality. The authors further state that the quality of the available evidence is inadequate due to short trial duration, selective reporting and publication bias, and they emphasize the paucity of data on suicidal ideation and behavior in antidepressant trials.

The issue of increased risk of suicidality with antidepressants was specifically addressed in two articles. In the first, Spielmans et al. review the scientific evidence and conclude that the FDA black-box suicidality warning was justified and firmly rooted in solid data from placebo-controlled antidepressant trials. They further detail that prominent claims suggesting that the FDA warning has led to decreasing prescription rates and thus increasing suicide rates were based on methodologically weak and potentially misleading ecological studies.

In the second, Whitely et al. describe how prominent psychiatrists and influential mental health organizations challenged the black-box suicidality warning for adolescents and young adults. The authors argue that various ecological studies were cited misleadingly as evidence that increased antidepressant use reduces youth suicide risk. Contrary to these claims, they further show that, in Australia, both antidepressant use and suicide rates increased substantially from 2008 to 2018.

Another serious safety issue was addressed by Kapra et al.. In their mini review they discuss the evidence for and against a potential effect of antidepressant use during pregnancy on autism spectrum disorders in the offspring. The authors found evidence for an association between prenatal SSRI exposure and an increased risk of autism spectrum disorders based on several observational studies, but caution that causality has not been demonstrated yet due to confounding by indication. The authors conclude that there is a need for more research on this serious safety issue, as accumulating data from animal studies suggest that SSRI exposure may alter normal brain development.

Trends of increasing antidepressant use in young people were addressed in two articles. In the first, Zito et al. analyzed administrative claims of Medicaid-insured youth aged <20 years from 1987 to 2014. During this 28-year period, antidepressant use grew 14-fold. They further show that in 2014, antidepressants were prescribed six times more often for youth in foster care than for their income-eligible Medicaid-counterparts. Off-label prescribing was also very common: a quarter of antidepressant-medicated youth were diagnosed with a behavioral disorder.

In the second, Cosgrove et al. state that antidepressant use in children and adolescents rose substantially over the last 15 years in part due to commercially driven off-label prescriptions, despite ongoing controversy over their effectiveness and safety. From the perspective of institutional corruption, they discuss two drivers of overuse resulting from an increasingly medicalised approach to mental health. The first is the empirically unsupported demand for depression screenings in youth and the second the emphasis on scaling up diagnosis and treatment of mental disorders as part of a renewed Global Mental Health Movement.

Last but not least, Locher et al. make an interesting case for open-label placebos in the treatment of chronic pain conditions in children and adolescents as an alternative to long-term antidepressant use. The authors acknowledge that this approach still lacks empirical evidence, but also stress that open-label placebos constitute a promising avenue for future research as they may help to mitigate the serious adverse effects of antidepressants.

In conclusion, the articles in this special topic demonstrate that pediatric antidepressant use is still controversial. Although antidepressant use in children and adolescents has increased substantially over the last 10–15 years, convincing evidence that the benefits outweigh the risks is lacking and treatment-emergent suicidality remains a major concern. Overuse and off-label prescribing are pressing issues, and there certainly is a need for safer and more effective treatments, both pharmacological and psychological (17). It is hoped that this article collection will spur innovative research and critical discussion.

## Author Contributions

The author confirms being the sole contributor of this work and has approved it for publication.

## Conflict of Interest

The author declares that the research was conducted in the absence of any commercial or financial relationships that could be construed as a potential conflict of interest.
